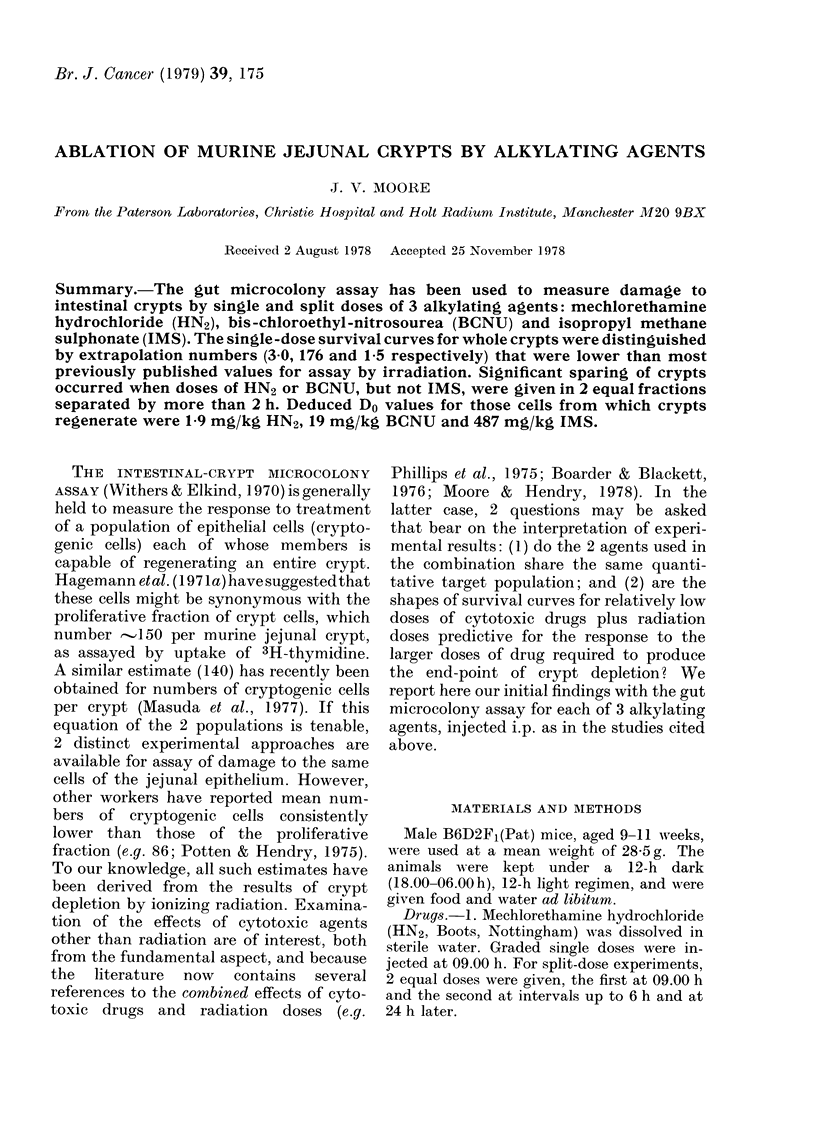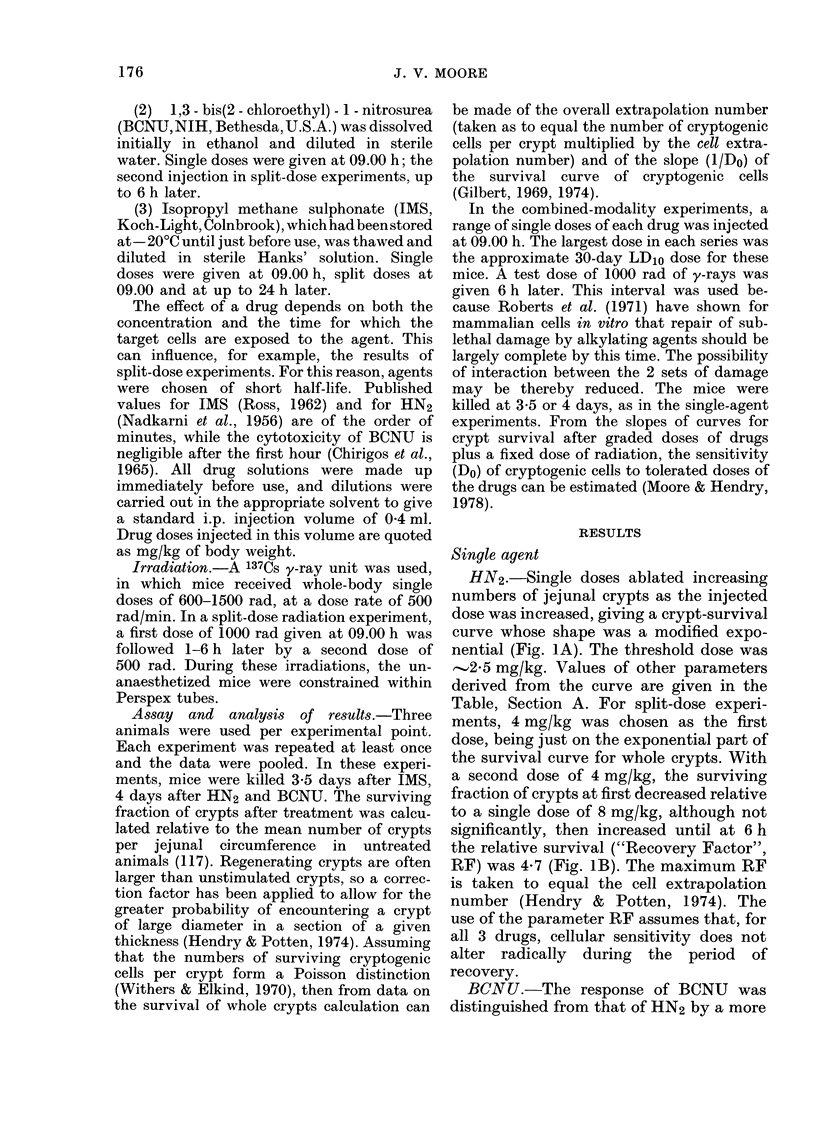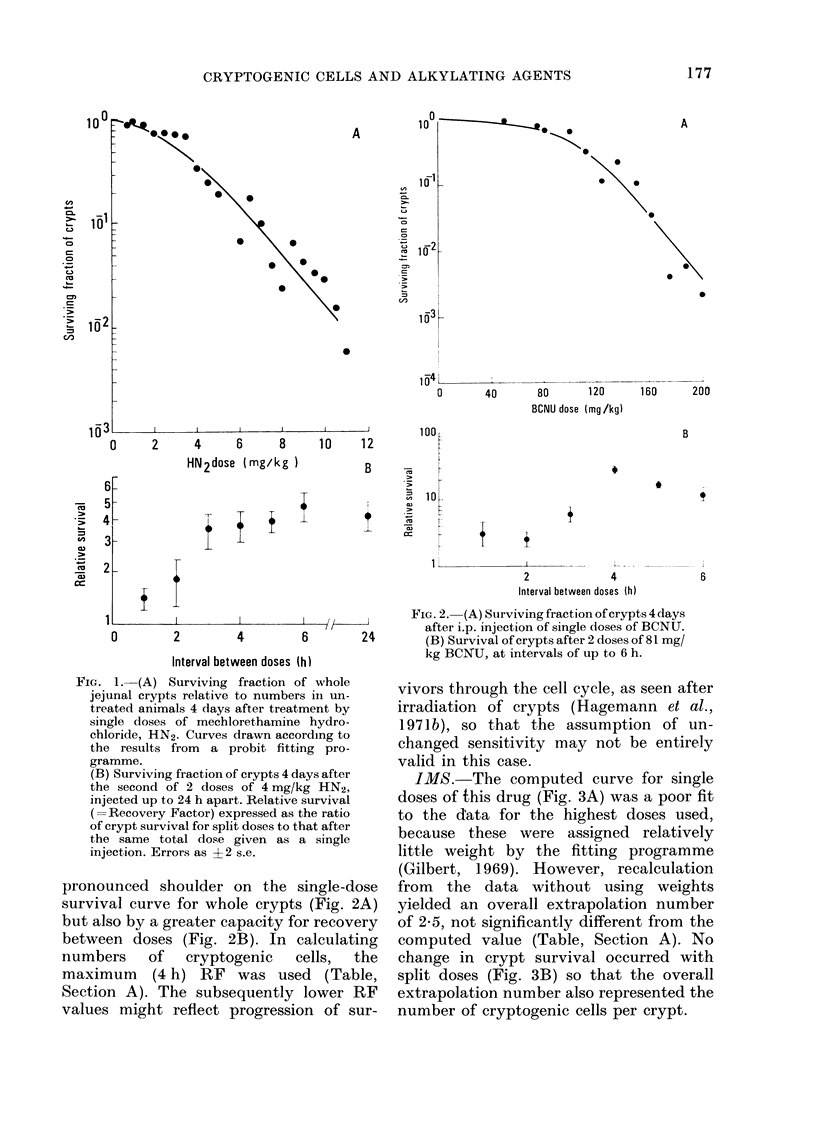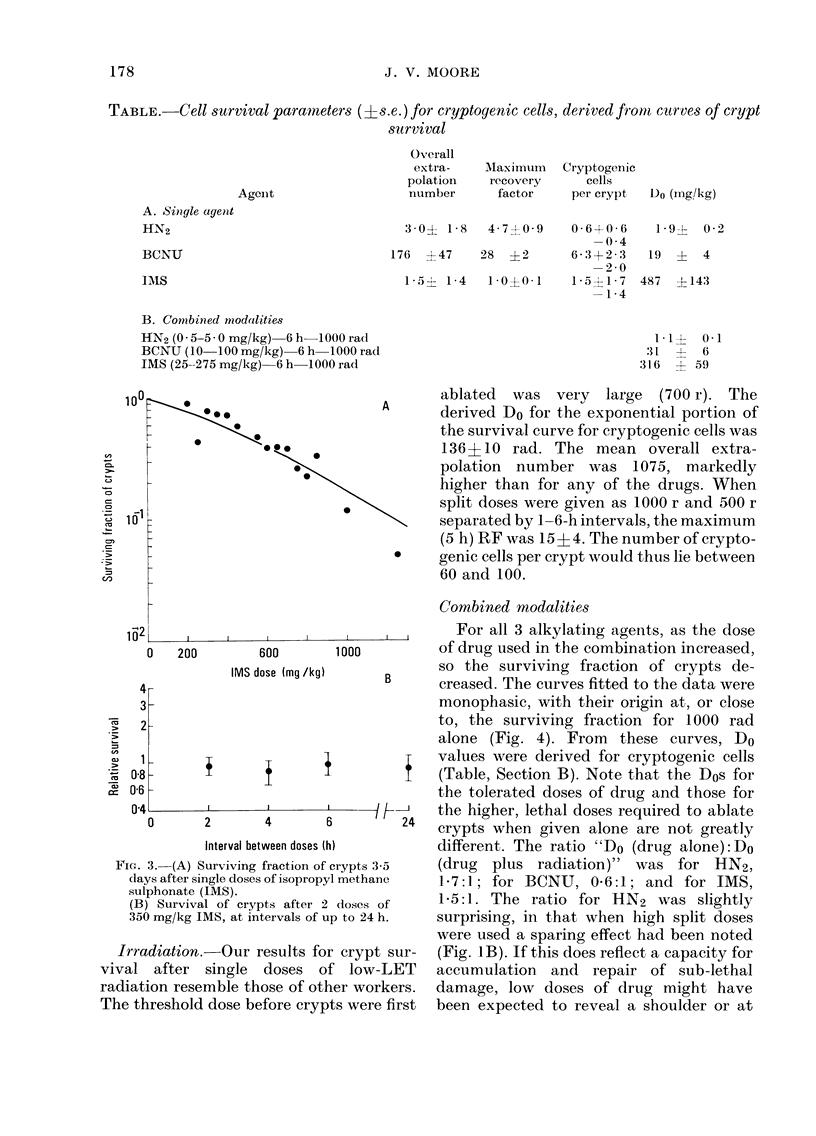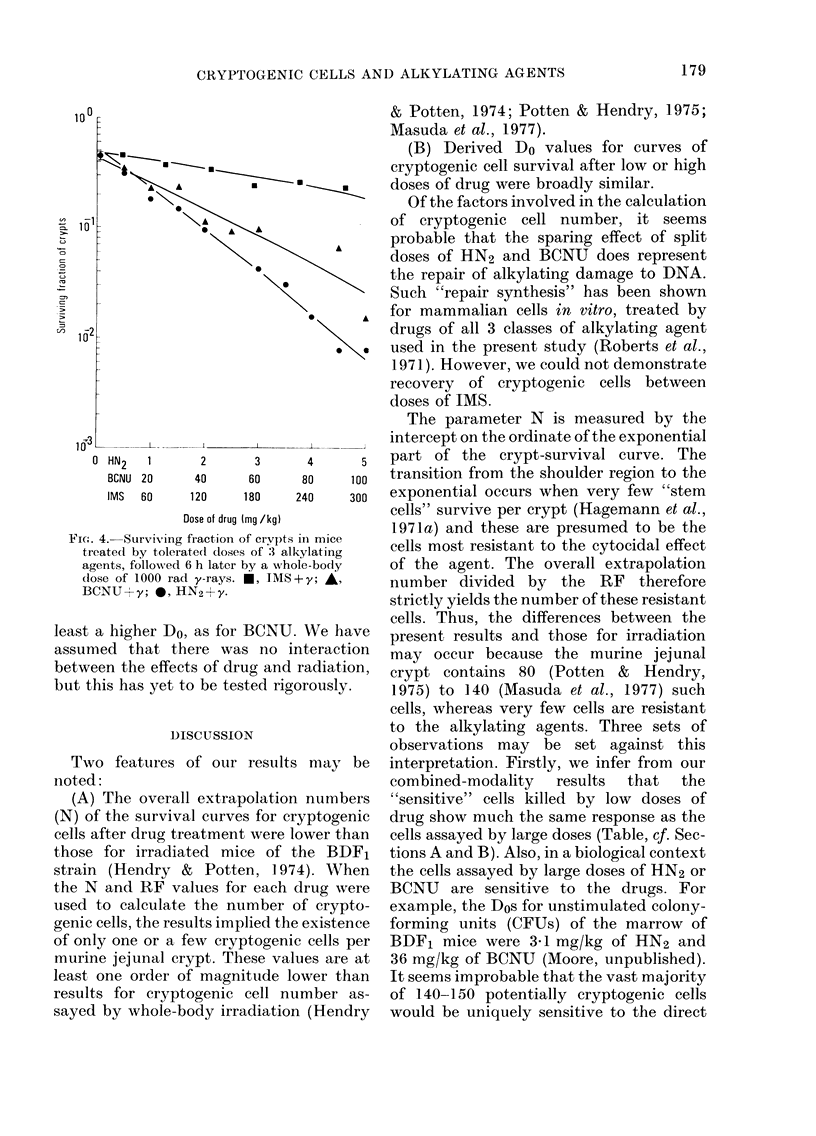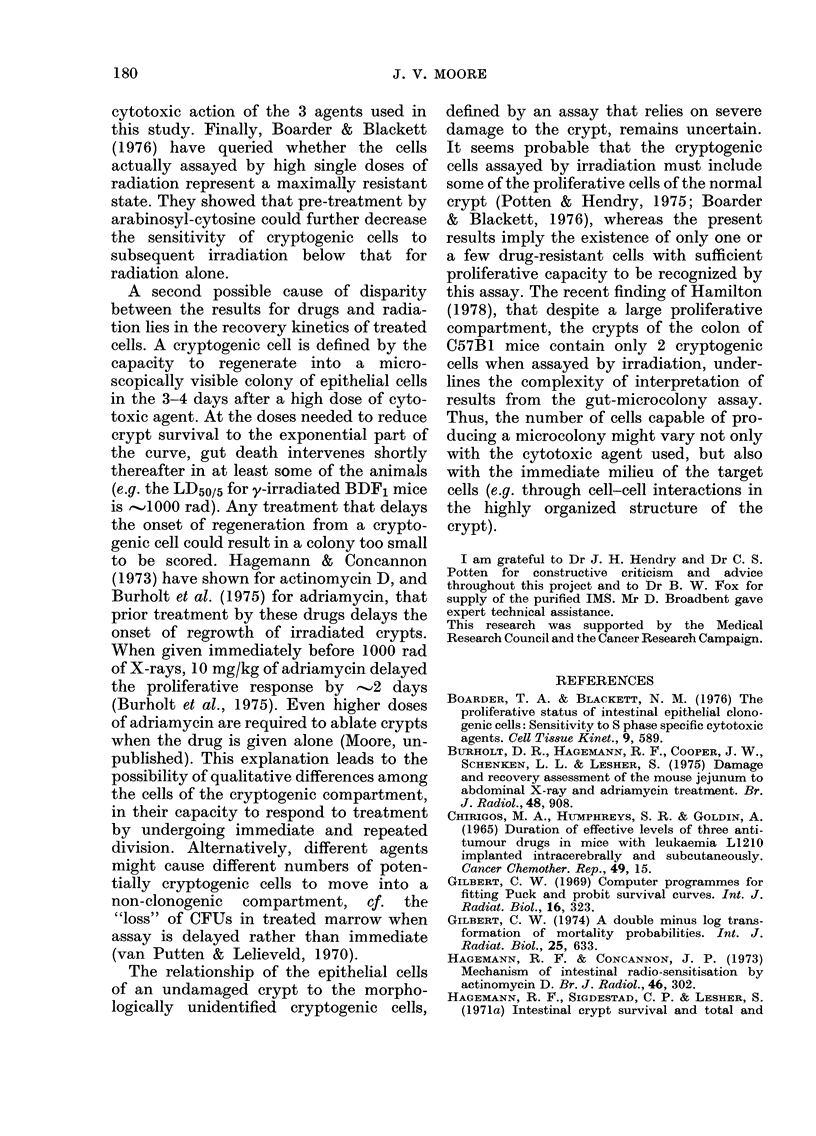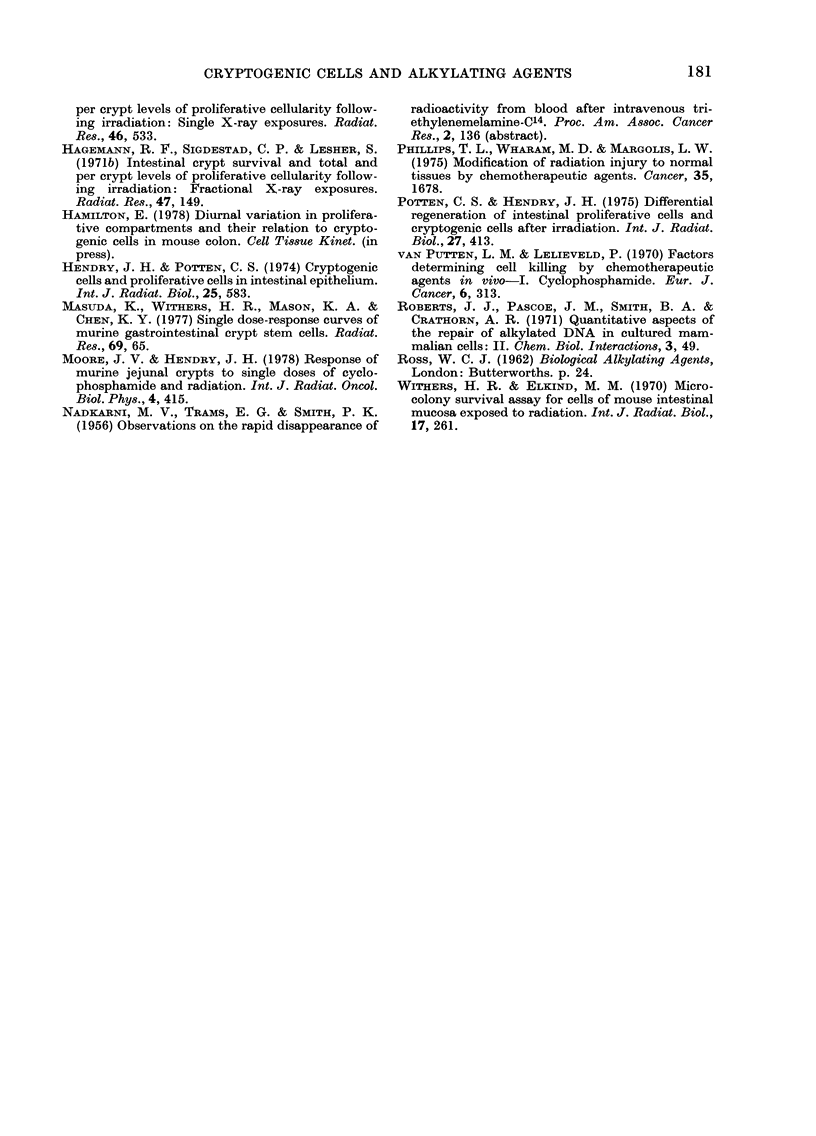# Ablation of murine jejunal crypts by alkylating agents.

**DOI:** 10.1038/bjc.1979.28

**Published:** 1979-02

**Authors:** J. V. Moore

## Abstract

The gut microcolony assay has been used to measure damage to intestinal crypts by single and split doses of 3 alkylating agents: mechlorethamine hydrochloride (HN2), bis-chloroethyl-nitrosourea (BCNU) and isopropyl methane sulphonate (IMS). The single-dose survival curves for whole crypts were distinguished by extrapolation numbers (3.0, 176 and 1.5 respectively) that were lower than most previously published values for assay by irradiation. Significant sparing of crypts occurred when doses of HN2 or BCNU, but not IMS, were given in 2 equal fractions separated by more than 2 h. Deduced D0 values for those cells from which crypts regenerate were 1.9 mg/kg HN2, 19 mg/kg BCNU and 487 mg/kg IMS.


*Br. J. Cancer* (1979) **39**, 175

### ABLATION OF MURINE JEJUNAL CRYPTS BY ALKYLATING AGENTS

J. V. MOORE

*From the Paterson Laboratories, Christie Hospital and Holt Radium Institute, Manchester M20 9BX*

Received 2 August 1978 Accepted 25 November 1978

**Summary.—The gut microcolony assay has been used to measure damage to intestinal crypts by single and split doses of 3 alkylating agents: mechlorethamine hydrochloride ($HN_2$), bis-chloroethyl-nitrosourea (BCNU) and isopropyl methane sulphonate (IMS). The single-dose survival curves for whole crypts were distinguished by extrapolation numbers (3·0, 176 and 1·5 respectively) that were lower than most previously published values for assay by irradiation. Significant sparing of crypts occurred when doses of $HN_2$ or BCNU, but not IMS, were given in 2 equal fractions separated by more than 2 h. Deduced $D_0$ values for those cells from which crypts regenerate were 1·9 mg/kg $HN_2$, 19 mg/kg BCNU and 487 mg/kg IMS.**

THE INTESTINAL-CRYPT MICROCOLONY ASSAY (Withers & Elkind, 1970) is generally held to measure the response to treatment of a population of epithelial cells (cryptogenic cells) each of whose members is capable of regenerating an entire crypt. Hagemann *et al.* (1971*a*) have suggested that these cells might be synonymous with the proliferative fraction of crypt cells, which number ~150 per murine jejunal crypt, as assayed by uptake of $^3$H-thymidine. A similar estimate (140) has recently been obtained for numbers of cryptogenic cells per crypt (Masuda *et al.*, 1977). If this equation of the 2 populations is tenable, 2 distinct experimental approaches are available for assay of damage to the same cells of the jejunal epithelium. However, other workers have reported mean numbers of cryptogenic cells consistently lower than those of the proliferative fraction (*e.g.* 86; Potten & Hendry, 1975). To our knowledge, all such estimates have been derived from the results of crypt depletion by ionizing radiation. Examination of the effects of cytotoxic agents other than radiation are of interest, both from the fundamental aspect, and because the literature now contains several references to the *combined* effects of cytotoxic drugs and radiation doses (*e.g.* Phillips *et al.*, 1975; Boarder & Blackett, 1976; Moore & Hendry, 1978). In the latter case, 2 questions may be asked that bear on the interpretation of experimental results: (1) do the 2 agents used in the combination share the same quantitative target population; and (2) are the shapes of survival curves for relatively low doses of cytotoxic drugs plus radiation doses predictive for the response to the larger doses of drug required to produce the end-point of crypt depletion? We report here our initial findings with the gut microcolony assay for each of 3 alkylating agents, injected i.p. as in the studies cited above.

### MATERIALS AND METHODS

Male B6D2F$_1$(Pat) mice, aged 9–11 weeks, were used at a mean weight of 28·5 g. The animals were kept under a 12-h dark (18.00–06.00 h), 12-h light regimen, and were given food and water *ad libitum*.

*Drugs*.—1. Mechlorethamine hydrochloride ($HN_2$, Boots, Nottingham) was dissolved in sterile water. Graded single doses were injected at 09.00 h. For split-dose experiments, 2 equal doses were given, the first at 09.00 h and the second at intervals up to 6 h and at 24 h later.

(2) 1,3 - bis(2 - chloroethyl) - 1 - nitrosurea (BCNU, NIH, Bethesda, U.S.A.) was dissolved initially in ethanol and diluted in sterile water. Single doses were given at 09.00 h; the second injection in split-dose experiments, up to 6 h later.

(3) Isopropyl methane sulphonate (IMS, Koch-Light, Colnbrook), which had been stored at $-20°C$ until just before use, was thawed and diluted in sterile Hanks' solution. Single doses were given at 09.00 h, split doses at 09.00 and at up to 24 h later.

The effect of a drug depends on both the concentration and the time for which the target cells are exposed to the agent. This can influence, for example, the results of split-dose experiments. For this reason, agents were chosen of short half-life. Published values for IMS (Ross, 1962) and for $HN_2$ (Nadkarni *et al.*, 1956) are of the order of minutes, while the cytotoxicity of BCNU is negligible after the first hour (Chirigos *et al.*, 1965). All drug solutions were made up immediately before use, and dilutions were carried out in the appropriate solvent to give a standard i.p. injection volume of 0·4 ml. Drug doses injected in this volume are quoted as mg/kg of body weight.

*Irradiation.*—A $^{137}Cs$ $\gamma$-ray unit was used, in which mice received whole-body single doses of 600–1500 rad, at a dose rate of 500 rad/min. In a split-dose radiation experiment, a first dose of 1000 rad given at 09.00 h was followed 1–6 h later by a second dose of 500 rad. During these irradiations, the unanaesthetized mice were constrained within Perspex tubes.

*Assay and analysis of results.*—Three animals were used per experimental point. Each experiment was repeated at least once and the data were pooled. In these experiments, mice were killed 3·5 days after IMS, 4 days after $HN_2$ and BCNU. The surviving fraction of crypts after treatment was calculated relative to the mean number of crypts per jejunal circumference in untreated animals (117). Regenerating crypts are often larger than unstimulated crypts, so a correction factor has been applied to allow for the greater probability of encountering a crypt of large diameter in a section of a given thickness (Hendry & Potten, 1974). Assuming that the numbers of surviving cryptogenic cells per crypt form a Poisson distinction (Withers & Elkind, 1970), then from data on the survival of whole crypts calculation can be made of the overall extrapolation number (taken as to equal the number of cryptogenic cells per crypt multiplied by the *cell* extrapolation number) and of the slope ($1/D_0$) of the survival curve of cryptogenic cells (Gilbert, 1969, 1974).

In the combined-modality experiments, a range of single doses of each drug was injected at 09.00 h. The largest dose in each series was the approximate 30-day $LD_{10}$ dose for these mice. A test dose of 1000 rad of $\gamma$-rays was given 6 h later. This interval was used because Roberts *et al.* (1971) have shown for mammalian cells *in vitro* that repair of sublethal damage by alkylating agents should be largely complete by this time. The possibility of interaction between the 2 sets of damage may be thereby reduced. The mice were killed at 3·5 or 4 days, as in the single-agent experiments. From the slopes of curves for crypt survival after graded doses of drugs plus a fixed dose of radiation, the sensitivity ($D_0$) of cryptogenic cells to tolerated doses of the drugs can be estimated (Moore & Hendry, 1978).

### RESULTS

### *Single agent*

*$HN_2$.*—Single doses ablated increasing numbers of jejunal crypts as the injected dose was increased, giving a crypt-survival curve whose shape was a modified exponential (Fig. 1A). The threshold dose was $\sim$2·5 mg/kg. Values of other parameters derived from the curve are given in the Table, Section A. For split-dose experiments, 4 mg/kg was chosen as the first dose, being just on the exponential part of the survival curve for whole crypts. With a second dose of 4 mg/kg, the surviving fraction of crypts at first decreased relative to a single dose of 8 mg/kg, although not significantly, then increased until at 6 h the relative survival ("Recovery Factor", RF) was 4·7 (Fig. 1B). The maximum RF is taken to equal the cell extrapolation number (Hendry & Potten, 1974). The use of the parameter RF assumes that, for all 3 drugs, cellular sensitivity does not alter radically during the period of recovery.

*BCNU.*—The response of BCNU was distinguished from that of $HN_2$ by a more

FIG. 1.—(A) Surviving fraction of whole jejunal crypts relative to numbers in untreated animals 4 days after treatment by single doses of mechlorethamine hydrochloride, $HN_2$. Curves drawn according to the results from a probit fitting programme.
(B) Surviving fraction of crypts 4 days after the second of 2 doses of 4 mg/kg $HN_2$, injected up to 24 h apart. Relative survival (=Recovery Factor) expressed as the ratio of crypt survival for split doses to that after the same total dose given as a single injection. Errors as $\pm 2$ s.e.

FIG. 2.—(A) Surviving fraction of crypts 4 days after i.p. injection of single doses of BCNU. (B) Survival of crypts after 2 doses of 81 mg/kg BCNU, at intervals of up to 6 h.

pronounced shoulder on the single-dose survival curve for whole crypts (Fig. 2A) but also by a greater capacity for recovery between doses (Fig. 2B). In calculating numbers of cryptogenic cells, the maximum (4 h) RF was used (Table, Section A). The subsequently lower RF values might reflect progression of survivors through the cell cycle, as seen after irradiation of crypts (Hagemann *et al.*, 1971*b*), so that the assumption of unchanged sensitivity may not be entirely valid in this case.

*IMS*.—The computed curve for single doses of this drug (Fig. 3A) was a poor fit to the data for the highest doses used, because these were assigned relatively little weight by the fitting programme (Gilbert, 1969). However, recalculation from the data without using weights yielded an overall extrapolation number of 2·5, not significantly different from the computed value (Table, Section A). No change in crypt survival occurred with split doses (Fig. 3B) so that the overall extrapolation number also represented the number of cryptogenic cells per crypt.

TABLE.—*Cell survival parameters* ($\pm$*s.e.*) *for cryptogenic cells, derived from curves of crypt survival*

| Agent | Overall extra-polation number | Maximum recovery factor | Cryptogenic cells per crypt | $D_0$ (mg/kg) |
|---|---|---|---|---|
| A. *Single agent* | | | | |
| $HN_2$ | $3 \cdot 0 \pm 1 \cdot 8$ | $4 \cdot 7 \pm 0 \cdot 9$ | $0 \cdot 6 {+0 \cdot 6 \atop -0 \cdot 4}$ | $1 \cdot 9 \pm 0 \cdot 2$ |
| BCNU | $176 \pm 47$ | $28 \pm 2$ | $6 \cdot 3 {+2 \cdot 3 \atop -2 \cdot 0}$ | $19 \pm 4$ |
| IMS | $1 \cdot 5 \pm 1 \cdot 4$ | $1 \cdot 0 \pm 0 \cdot 1$ | $1 \cdot 5 {+1 \cdot 7 \atop -1 \cdot 4}$ | $487 \pm 143$ |
| B. *Combined modalities* | | | | |
| $HN_2$ (0·5–5·0 mg/kg)—6 h—1000 rad | | | | $1 \cdot 1 \pm 0 \cdot 1$ |
| BCNU (10—100 mg/kg)—6 h—1000 rad | | | | $31 \pm 6$ |
| IMS (25–275 mg/kg)—6 h—1000 rad | | | | $316 \pm 59$ |

FIG. 3.—(A) Surviving fraction of crypts 3·5 days after single doses of isopropyl methane sulphonate (IMS).
(B) Survival of crypts after 2 doses of 350 mg/kg IMS, at intervals of up to 24 h.

*Irradiation*.—Our results for crypt survival after single doses of low-LET radiation resemble those of other workers. The threshold dose before crypts were first ablated was very large (700 r). The derived $D_0$ for the exponential portion of the survival curve for cryptogenic cells was $136 \pm 10$ rad. The mean overall extrapolation number was 1075, markedly higher than for any of the drugs. When split doses were given as 1000 r and 500 r separated by 1–6-h intervals, the maximum (5 h) RF was $15 \pm 4$. The number of cryptogenic cells per crypt would thus lie between 60 and 100.

### *Combined modalities*

For all 3 alkylating agents, as the dose of drug used in the combination increased, so the surviving fraction of crypts decreased. The curves fitted to the data were monophasic, with their origin at, or close to, the surviving fraction for 1000 rad alone (Fig. 4). From these curves, $D_0$ values were derived for cryptogenic cells (Table, Section B). Note that the $D_0$s for the tolerated doses of drug and those for the higher, lethal doses required to ablate crypts when given alone are not greatly different. The ratio "$D_0$ (drug alone):$D_0$ (drug plus radiation)" was for $HN_2$, 1·7:1; for BCNU, 0·6:1; and for IMS, 1·5:1. The ratio for $HN_2$ was slightly surprising, in that when high split doses were used a sparing effect had been noted (Fig. 1B). If this does reflect a capacity for accumulation and repair of sub-lethal damage, low doses of drug might have been expected to reveal a shoulder or at

FIG. 4.—Surviving fraction of crypts in mice treated by tolerated doses of 3 alkylating agents, followed 6 h later by a whole-body dose of 1000 rad γ-rays. ■, IMS+γ; ▲, BCNU+γ; ●, $HN_2$+γ.

least a higher $D_0$, as for BCNU. We have assumed that there was no interaction between the effects of drug and radiation, but this has yet to be tested rigorously.

### DISCUSSION

Two features of our results may be noted:

(A) The overall extrapolation numbers (N) of the survival curves for cryptogenic cells after drug treatment were lower than those for irradiated mice of the $BDF_1$ strain (Hendry & Potten, 1974). When the N and RF values for each drug were used to calculate the number of cryptogenic cells, the results implied the existence of only one or a few cryptogenic cells per murine jejunal crypt. These values are at least one order of magnitude lower than results for cryptogenic cell number assayed by whole-body irradiation (Hendry & Potten, 1974; Potten & Hendry, 1975; Masuda *et al.*, 1977).

(B) Derived $D_0$ values for curves of cryptogenic cell survival after low or high doses of drug were broadly similar.

Of the factors involved in the calculation of cryptogenic cell number, it seems probable that the sparing effect of split doses of $HN_2$ and BCNU does represent the repair of alkylating damage to DNA. Such "repair synthesis" has been shown for mammalian cells *in vitro*, treated by drugs of all 3 classes of alkylating agent used in the present study (Roberts *et al.*, 1971). However, we could not demonstrate recovery of cryptogenic cells between doses of IMS.

The parameter N is measured by the intercept on the ordinate of the exponential part of the crypt-survival curve. The transition from the shoulder region to the exponential occurs when very few "stem cells" survive per crypt (Hagemann *et al.*, 1971*a*) and these are presumed to be the cells most resistant to the cytocidal effect of the agent. The overall extrapolation number divided by the RF therefore strictly yields the number of these resistant cells. Thus, the differences between the present results and those for irradiation may occur because the murine jejunal crypt contains 80 (Potten & Hendry, 1975) to 140 (Masuda *et al.*, 1977) such cells, whereas very few cells are resistant to the alkylating agents. Three sets of observations may be set against this interpretation. Firstly, we infer from our combined-modality results that the "sensitive" cells killed by low doses of drug show much the same response as the cells assayed by large doses (Table, *cf.* Sections A and B). Also, in a biological context the cells assayed by large doses of $HN_2$ or BCNU are sensitive to the drugs. For example, the $D_0$s for unstimulated colony-forming units (CFUs) of the marrow of $BDF_1$ mice were 3·1 mg/kg of $HN_2$ and 36 mg/kg of BCNU (Moore, unpublished). It seems improbable that the vast majority of 140–150 potentially cryptogenic cells would be uniquely sensitive to the direct cytotoxic action of the 3 agents used in this study. Finally, Boarder & Blackett (1976) have queried whether the cells actually assayed by high single doses of radiation represent a maximally resistant state. They showed that pre-treatment by arabinosyl-cytosine could further decrease the sensitivity of cryptogenic cells to subsequent irradiation below that for radiation alone.

A second possible cause of disparity between the results for drugs and radiation lies in the recovery kinetics of treated cells. A cryptogenic cell is defined by the capacity to regenerate into a microscopically visible colony of epithelial cells in the 3–4 days after a high dose of cytotoxic agent. At the doses needed to reduce crypt survival to the exponential part of the curve, gut death intervenes shortly thereafter in at least some of the animals (*e.g.* the $LD_{50/5}$ for $\gamma$-irradiated $BDF_1$ mice is $\sim$1000 rad). Any treatment that delays the onset of regeneration from a cryptogenic cell could result in a colony too small to be scored. Hagemann & Concannon (1973) have shown for actinomycin D, and Burholt *et al.* (1975) for adriamycin, that prior treatment by these drugs delays the onset of regrowth of irradiated crypts. When given immediately before 1000 rad of X-rays, 10 mg/kg of adriamycin delayed the proliferative response by $\sim$2 days (Burholt *et al.*, 1975). Even higher doses of adriamycin are required to ablate crypts when the drug is given alone (Moore, unpublished). This explanation leads to the possibility of qualitative differences among the cells of the cryptogenic compartment, in their capacity to respond to treatment by undergoing immediate and repeated division. Alternatively, different agents might cause different numbers of potentially cryptogenic cells to move into a non-clonogenic compartment, *cf.* the "loss" of CFUs in treated marrow when assay is delayed rather than immediate (van Putten & Lelieveld, 1970).

The relationship of the epithelial cells of an undamaged crypt to the morphologically unidentified cryptogenic cells, defined by an assay that relies on severe damage to the crypt, remains uncertain. It seems probable that the cryptogenic cells assayed by irradiation must include some of the proliferative cells of the normal crypt (Potten & Hendry, 1975; Boarder & Blackett, 1976), whereas the present results imply the existence of only one or a few drug-resistant cells with sufficient proliferative capacity to be recognized by this assay. The recent finding of Hamilton (1978), that despite a large proliferative compartment, the crypts of the colon of C57Bl mice contain only 2 cryptogenic cells when assayed by irradiation, underlines the complexity of interpretation of results from the gut-microcolony assay. Thus, the number of cells capable of producing a microcolony might vary not only with the cytotoxic agent used, but also with the immediate milieu of the target cells (*e.g.* through cell–cell interactions in the highly organized structure of the crypt).

I am grateful to Dr J. H. Hendry and Dr C. S. Potten for constructive criticism and advice throughout this project and to Dr B. W. Fox for supply of the purified IMS. Mr D. Broadbent gave expert technical assistance.

This research was supported by the Medical Research Council and the Cancer Research Campaign.

### REFERENCES

Boarder, T. A. & Blackett, N. M. (1976) The proliferative status of intestinal epithelial clonogenic cells: Sensitivity to S phase specific cytotoxic agents. *Cell Tissue Kinet.*, **9**, 589.

Burholt, D. R., Hagemann, R. F., Cooper, J. W., Schenken, L. L. & Lesher, S. (1975) Damage and recovery assessment of the mouse jejunum to abdominal X-ray and adriamycin treatment. *Br. J. Radiol.*, **48**, 908.

Chirigos, M. A., Humphreys, S. R. & Goldin, A. (1965) Duration of effective levels of three antitumour drugs in mice with leukaemia L1210 implanted intracerebrally and subcutaneously. *Cancer Chemother. Rep.*, **49**, 15.

Gilbert, C. W. (1969) Computer programmes for fitting Puck and probit survival curves. *Int. J. Radiat. Biol.*, **16**, 323.

Gilbert, C. W. (1974) A double minus log transformation of mortality probabilities. *Int. J. Radiat. Biol.*, **25**, 633.

Hagemann, R. F. & Concannon, J. P. (1973) Mechanism of intestinal radio-sensitisation by actinomycin D. *Br. J. Radiol.*, **46**, 302.

Hagemann, R. F., Sigdestad, C. P. & Lesher, S. (1971*a*) Intestinal crypt survival and total and per crypt levels of proliferative cellularity following irradiation: Single X-ray exposures. *Radiat. Res.*, **46**, 533.

HAGEMANN, R. F., SIGDESTAD, C. P. & LESHER, S. (1971*b*) Intestinal crypt survival and total and per crypt levels of proliferative cellularity following irradiation: Fractional X-ray exposures. *Radiat. Res.*, **47**, 149.

HAMILTON, E. (1978) Diurnal variation in proliferative compartments and their relation to cryptogenic cells in mouse colon. *Cell Tissue Kinet.* (in press).

HENDRY, J. H. & POTTEN, C. S. (1974) Cryptogenic cells and proliferative cells in intestinal epithelium. *Int. J. Radiat. Biol.*, **25**, 583.

MASUDA, K., WITHERS, H. R., MASON, K. A. & CHEN, K. Y. (1977) Single dose-response curves of murine gastrointestinal crypt stem cells. *Radiat. Res.*, **69**, 65.

MOORE, J. V. & HENDRY, J. H. (1978) Response of murine jejunal crypts to single doses of cyclophosphamide and radiation. *Int. J. Radiat. Oncol. Biol. Phys.*, **4**, 415.

NADKARNI, M. V., TRAMS, E. G. & SMITH, P. K. (1956) Observations on the rapid disappearance of radioactivity from blood after intravenous triethylenemelamine-$C^{14}$. *Proc. Am. Assoc. Cancer Res.*, **2**, 136 (abstract).

PHILLIPS, T. L., WHARAM, M. D. & MARGOLIS, L. W. (1975) Modification of radiation injury to normal tissues by chemotherapeutic agents. *Cancer*, **35**, 1678.

POTTEN, C. S. & HENDRY, J. H. (1975) Differential regeneration of intestinal proliferative cells and cryptogenic cells after irradiation. *Int. J. Radiat. Biol.*, **27**, 413.

VAN PUTTEN, L. M. & LELIEVELD, P. (1970) Factors determining cell killing by chemotherapeutic agents *in vivo*—I. Cyclophosphamide. *Eur. J. Cancer*, **6**, 313.

ROBERTS, J. J., PASCOE, J. M., SMITH, B. A. & CRATHORN, A. R. (1971) Quantitative aspects of the repair of alkylated DNA in cultured mammalian cells: II. *Chem. Biol. Interactions*, **3**, 49.

ROSS, W. C. J. (1962) *Biological Alkylating Agents*, London: Butterworths. p. 24.

WITHERS, H. R. & ELKIND, M. M. (1970) Microcolony survival assay for cells of mouse intestinal mucosa exposed to radiation. *Int. J. Radiat. Biol.*, **17**, 261.